# Association between Dietary Quality and Non-Alcoholic Fatty Liver Disease in Korean Adults: A Nationwide, Population-Based Study Using the Korean Healthy Eating Index (2013–2021)

**DOI:** 10.3390/nu16101516

**Published:** 2024-05-17

**Authors:** Seong-Uk Baek, Taeyeon Kim, Yu-Min Lee, Jong-Uk Won, Jin-Ha Yoon

**Affiliations:** 1 Department of Occupational and Environmental Medicine, Severance Hospital, Yonsei University College of Medicine, Seoul 03722, Republic of Korea; 2 The Institute for Occupational Health, Yonsei University College of Medicine, Seoul 03722, Republic of Korea; 3 Graduate School, Yonsei University College of Medicine, Seoul 03722, Republic of Korea; 4 Graduate School of Public Health, Yonsei University College of Medicine, Seoul 03722, Republic of Korea; 5 Department of Preventive Medicine, Yonsei University College of Medicine, Seoul 03722, Republic of Korea

**Keywords:** fatty liver disease, healthy eating index, healthy diet, healthy eating, hepatic steatosis

## Abstract

This study explored the relationship between the Korean Healthy Eating Index (KHEI) and non-alcoholic fatty liver disease (NAFLD). This cross-sectional study included 34,174 Korean adults. The KHEI was composed of three subcomponents (adequacy, moderation, and energy balance) and calculated based on a 24 h dietary recall. The total score ranged from 0 to 100, with a higher score indicating a greater adherence to the Korean dietary guidelines. The total KHEI scores were categorized into quartiles (Q1–Q4). NAFLD was classified using the hepatic steatosis index. Logistic regressions were used to estimate odds ratios (ORs) and 95% confidence intervals (CIs). The adjusted OR (95% CI) of NAFLD was 0.95 (0.87–1.03) for the Q2 group, 0.90 (0.83–0.98) for the Q3 group, and 0.79 (0.72–0.87) for the Q4 group, compared with the Q1 group. Among the subcomponents of the KHEI, individuals with high scores in the adequacy component, characterized by an abundant consumption of fruits, vegetables, and dairy products, exhibited the most pronounced association with NAFLD. A higher KHEI score was negatively associated with NAFLD in Korean adults. Therefore, the promotion of healthy dietary patterns can play a beneficial role in the prevention or management of NAFLD.

## 1. Introduction

Non-alcoholic fatty liver disease (NAFLD), characterized by the accumulation of excess fat in hepatic cells as a manifestation of metabolic dysfunction, has emerged as a significant public health concern. Along with an increase in the incidence of obesity and metabolic syndrome, the incidence of NAFLD and its associated health burden has been growing recently [1]. A parallel trend has been noted in Korea, with the prevalence of NAFLD steadily increasing since 2010. In Korea, approximately 15.5% of adults have NAFLD; this upward trajectory is pronounced among young adults, particularly males [2].

A healthy diet is a crucial element in the prevention and management of NAFLD. Previous studies have found that an increased consumption of saturated fatty acids and carbohydrates and a reduced consumption of dietary fiber were associated with NAFLD [3,4,5,6,7]. Recently, there has been a growing research interest in approaches that emphasize dietary patterns over individual nutrients or foods, taking a comprehensive view of overall diet quality. These approaches are capable of capturing real-world dietary practices and incorporating the interactive or cumulative impact of nutrients or food components on health outcomes [8,9]. Recent epidemiological studies have shown that healthy dietary patterns, often measured by the Healthy Eating Index, are associated with a reduced risk of fatty liver disease [10,11,12,13]. Previous studies have suggested that healthy dietary patterns can contribute to a decreased risk of NAFLD by enhancing one’s metabolic status, including maintaining a healthy body weight and improving insulin resistance [14,15].

Although previous studies have found that healthy dietary patterns are associated with a reduced risk and prevalence of NAFLD, they were limited by the fact that most concentrated on Western populations, and the indices or scoring systems used to assess dietary patterns were developed within the context of Western cultures. Marked variations exist between cultures in the consumption patterns of foods and nutrients and in dietary habits, which can lead to different health outcomes. Korean dietary habits in particular are characterized by a high consumption of salted dishes and abundant quantities of vegetables along with a low intake of milk or calcium [16]. The recently developed Korean Healthy Eating Index (KHEI), designed for assessing the dietary patterns of the Korean population and reflecting the National Dietary Guidelines for Koreans [17], has demonstrated an inverse association with various metabolic diseases, including obesity [18], metabolic syndrome [19], and hypertriglyceridemia [20], which are well-documented cardiometabolic risk factors for NAFLD. Nonetheless, to the best of our knowledge, the association between the KHEI and NAFLD has not been explored in the literature. Therefore, we aimed to investigate the association between the KHEI and NAFLD based on a nationally representative sample of the Korean population.

## 2. Materials and Methods

### 2.1. Study Population

The study sample was obtained from the sixth and eighth waves of the Korea National Health and Nutrition Examination Survey (KNHANES), conducted from 2013 to 2021 by the Korea Disease Control and Prevention Agency (KDCA). To select nationally representative samples of the Korean population, the KDCA employed a multi-stage clustered probability method, in which enumeration districts in Korea were selected as primary sampling units, and households in each region were selected as secondary sampling units [21]. The survey response rate was 81.7 and 80.8% for the sixth and seventh KNHANES surveys, respectively [22].

**Figure 1:**
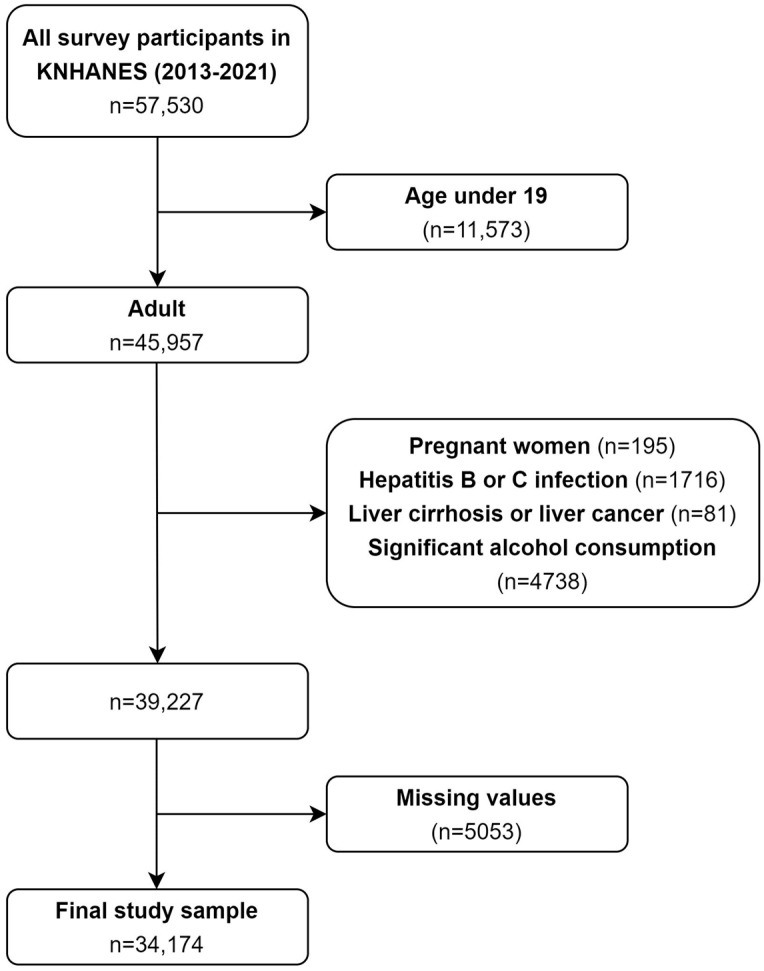
Flowchart of the selection process for the study sample.

The flowchart of the selection process for the final study sample is presented in Figure 1. Initially, we included adult survey respondents aged ≥19 years from the sixth and eighth waves of the KNHANES (2013–2021), when information on the healthy eating index was available. Subsequently, participants with any of the following characteristics, all of which have the potential to cause liver disease, were excluded [23]: current pregnancy; hepatitis B or C virus infection; history of liver cancer or cirrhosis; and history of significant alcohol consumption, defined as ≥210 g/week for men and 140 g/week for women. Additionally, observations with missing values were excluded. In total, 34,174 survey participants were included in the final sample.

### 2.2. Data Availability and Ethics Statement

The raw data of the KNHANES are available to the public and are accessible on the KNHANES website (https://knhanes.kdca.go.kr/knhanes, accessed on 23 December 2023) [21]. The KNHANES was conducted with the approval of the institutional review board of the KDCA (2013-07CON-03-4C; 2013-12EXP-03-5C; 2018-01-03-P-A; 2018-01-03-C-A; 2018-01-03-2C-A; 2018-01-03-5C-A). This secondary data analysis was approved with exempt status by the IRB of Yonsei Health System (IRB number 4-2023-0959, approval date 13 September 2023).

### 2.3. Variables

#### 2.3.1. Korean Healthy Eating Index

The main independent variable used in this study was dietary pattern, which was assessed by the KHEI. The KHEI was developed jointly by the KCDA and the Korean Nutrition Society [24]. Based on the National Dietary Guidelines for Koreans [17], the 14-item KHEI comprises the following components: adequacy, moderation, and energy balance. The adequacy component assesses whether the recommended consumption of food and nutrients is adequately met. It consists of the following eight items: eating breakfast; mixed grains; total fruits; fresh fruits; total vegetables; vegetables excluding kimchi and pickles; meat, fish, eggs, and beans; and milk and dairy products. The moderation component assesses the restricted intakes of foods and nutrients using three items: the percentage of energy from saturated fatty acids, sodium intake, and the percentage of energy from sweets and beverages. The balance component assesses the balance of energy intake and macronutrients using three items: the percentage of energy from carbohydrates, the percentage of energy from fats, and total energy intake. 

The detailed scoring system of the KHEI is described in Table S1. The scores for each item are calculated as continuous variables based on the amount and frequency of food or meal intake relative to the maximum criteria. Further details on the scoring system can be found in a previous study [24]. For items in the adequacy component, scores are assigned based on whether a certain level of serving for each food was consumed daily, with higher scores designed to reflect greater consumption. For breakfast intake, meat, fish, egg, and bean intake, and milk and milk product intake, scores range between 0 and 10 points. For the remaining items, scores range between 0 and 5 points. For items in the moderation component, scores are assigned based on whether a certain level of intake for each nutrient and food is consumed daily, with higher scores designed to reflect lower consumption. Each item corresponding to saturated fatty acid intake, sodium intake, and the consumption of sweets, beverages, and alcoholic drinks is scored on a scale from 0 to 10. For items in the energy balance component, we evaluated whether an adequate level of energy was consumed for each item, and scores were designed to maximize when energy intake from the respective nutrient fell within a specific range. Each item in the energy balance component is scored on a scale of 0 to 5.

The total KHEI scores range from 0 to 100, with a higher score indicating a better dietary pattern that meets the Korean guidelines for a healthy diet [17,24]. Information on dietary intake was assessed through a 24 h dietary recall method, and total energy and nutrient intake were calculated using the Standard Food Composition Table, eighth revision [25]. All participants were surveyed using home visits, with in-person interviews conducted by trained dieticians [24]. Survey respondents were categorized into quartiles based on their total score and subscores for the adequacy, moderation, and balance components of KHEI. More detailed information on each item of the KHEI can be found in a paper by Yun et al. (2020) [24].

#### 2.3.2. Non-Alcoholic Fatty Liver Disease

The presence of NAFLD was determined using the hepatic steatosis index (HSI), a validated tool for assessing fatty liver in the Korean population [26]. Developed for NAFLD screening in Koreans without the need for invasive procedures, the HSI utilizes serum alanine aminotransferase/aspartate aminotransferase (ALT/AST) ratio, body mass index, diabetes mellitus status, and sex as formula parameters.

$$HSI=8\times (ALT/AST)+BMI\;(kg/m^{2})+2\;(if\;diabetes\;mellitus)+2\;(if\;female)$$

Survey participants who met at least one of the following criteria were classified as having diabetes mellitus: glycated hemoglobin ≥6.5% mg/dL, fasting plasma glucose ≥126 mg/dL, and use of anti-diabetic oral agents or insulin. An HSI of ≥36 was defined as indicative of NAFLD in this study [26]. Previous research has demonstrated the HSI’s effectiveness in identifying NAFLD, with an area under the receiver operating characteristic ranging from 0.82 to 0.86 [26,27]. As a result, the HSI has become a widely used tool for epidemiological studies in Korea [2,28]. According to a previous study, the precision of using HSI > 36 for diagnosing fatty liver disease in a community sample in South Korea was as follows: the sensitivity was 50.5%, the specificity was 79.0%, the positive predictive value was 50.9%, and the negative predictive value was 78.7% [29].

As supplementary criteria, the K-NAFLD score [30] and the ZJU index [31] were employed for sensitivity analyses. The detailed information on these scoring systems is presented in Table S2.

#### 2.3.3. Confounders

Sex was adjusted. Age was categorized as <30, 30 to 39, 40 to 49, 50 to 59, and ≥60 years. Educational attainment was categorized as middle school or below, high school, and college or above. Income level was classified based on quartile values of total household income for each survey year (Q1–Q4). Marital status was categorized as married and unmarried/other. Employment status was categorized as employed, self-employed, and unemployed. Smoking status was categorized as current smoker and past/never smoker. Physical activity was categorized based on whether or not each survey participant engaged in ≥150 min/week of moderate-to-vigorous physical activity, in accordance with the current physical activity guidelines [32]. Additionally, survey years were adjusted for the regression analyses.

### 2.4. Statistical Analysis

For descriptive analyses, we investigated the characteristics of the survey participants according to their total KHEI scores. Next, we explored the prevalence of NAFLD according to the study variables. We then explored how the subcomponents of the KHEI differ by KHEI categories (Q1–Q4). For regression analyses, we first explored the association between categories by KHEI total score (Q1–Q4) and NAFLD status. Subsequently, we explored how NAFLD was associated with each subcomponent of the KHEI (adequacy, moderation, and balance). For sensitivity analyses, we first utilized supplementary criteria for NAFLD classification and checked the robustness of our findings. Additionally, we employed multiple imputation to handle missing values in our datasets. A total of 20 datasets were generated using multiple imputation by chained equations. Estimations were combined based on Rubin’s rule. Logistic regression models were fitted to estimate odds ratios (ORs) and their corresponding 95% confidence intervals (CIs). 

All statistical analyses and visualizations were performed using the R software (version 4.2.3; R Foundation for Statistical Computing, Vienna, Austria). The complex survey design and survey weights assigned for each survey participant were considered for all descriptive and regression analyses. The R package “survey” and its function “svyglm” were used for statistical analyses.

## 3. Results

A total of 34,174 individuals, consisting of 13,143 men and 21,031 women, were included in the study (Table 1). Individuals with the highest level of dietary quality were more likely to be women, older, have a higher income level, be married, non-smokers, and engage in the recommended level of physical activity. The distribution of KHEI scores in the study sample is depicted in Figure S1. The specific variances in the subcomponents of the KHEI based on overall dietary patterns are presented in Table S1. The group with the highest KHEI score had higher mean scores in the adequacy component for fruit, vegetable, meat, fish, egg, bean, and dairy product intakes, as well as in items related to the moderation and energy balance components.

The prevalence of NAFLD was 26.2, 25.1, 24.6, and 20.5% in the lowest (Q1), low (Q2), high (Q3), and highest (Q4) KHEI score groups, respectively (Table 2). Furthermore, the prevalence of NAFLD was elevated among individuals of male sex, those with a low educational level and a low income level, self-employed workers, current smokers, and those with insufficient levels of physical activity. 

In the fully adjusted model, the OR (95% CI) of NAFLD was 0.95 (0.87–1.03) for those with a low total KHEI score (Q2), 0.90 (0.83–0.98) for those with a high total KHEI score (Q3), and 0.79 (0.72–0.87) for those with the highest total KHEI score (Q4), compared to the group with the lowest KHEI score (Q1) (Table 3).

**Figure 2:**
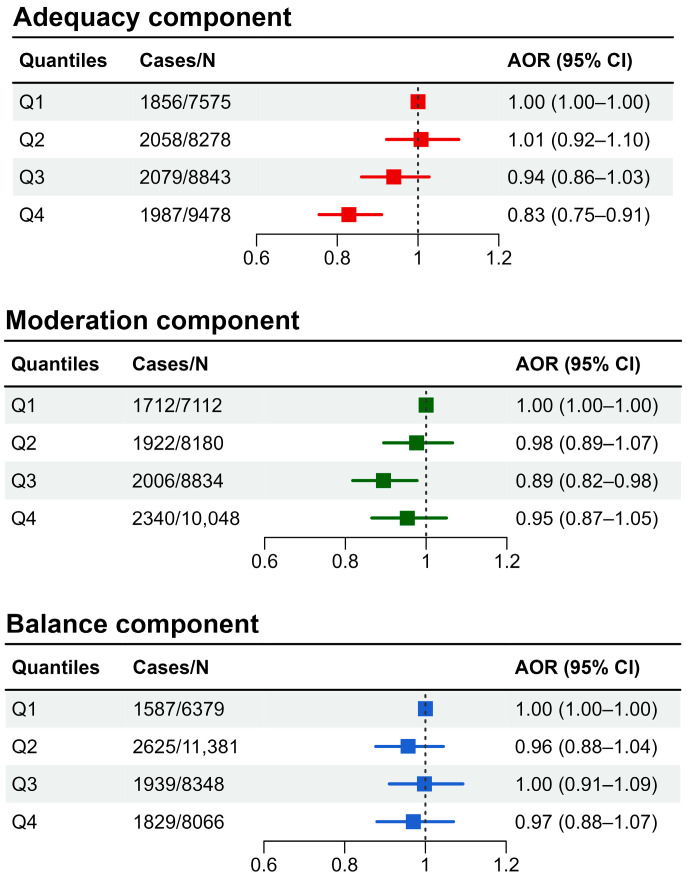
Association between subcomponents of Korean Healthy Eating Index and non-alcoholic fatty liver disease (AOR: adjusted odds ratio; CI: confidence interval).

The adjusted OR (95% CI) of the adequacy component and NAFLD was 1.01 (0.92–1.10) for the low group (Q2), 0.94 (0.86–1.03) for the high group (Q3), and 0.83 (0.75–0.91) for the highest group (Q4), compared to the lowest group (Q1) (Figure 2). The adjusted OR (95% CI) of the moderation component and NAFLD was 0.98 (0.89–1.07) for the low group (Q2), 0.89 (0.82–0.98) for the high group (Q3), and 0.95 (0.87–1.05) for the highest group (Q4), compared to the lowest group (Q1). The adjusted OR (95% CI) of the balance component and NAFLD was 0.96 (0.88–1.04) for the low group (Q2), 1.00 (0.91–1.09) for the high group (Q3), and 0.97 (0.88–1.07) for the highest group (Q4), compared to the lowest group (Q1).

The association between each of the 14 components of the KHEI and NAFLD is shown in Table S3. The results show that eating breakfast, a high consumption of fruits and milk, and a low consumption of sodium were negatively associated with NAFLD, while a high consumption of whole grains was positively associated with NAFLD.

The outcomes of sensitivity analyses employing alternative criteria for NAFLD classification are shown in Figures S2 and S3. A pattern comparable to the primary analysis emerged, wherein individuals with high total KHEI scores exhibited a reduced OR of NAFLD, and the adequacy component demonstrated a negative association with NAFLD. No clear positive or negative association between the moderation or balance components and NAFLD was observed in the sensitivity analyses. Sensitivity analyses conducted on the imputed datasets produced findings consistent with the primary analyses (Figure S4).

## 4. Discussion

This study was the first to investigate the relationship between Korean-style healthy dietary patterns and NAFLD in Korean adults. We observed that individuals with high KHEI scores were less likely to have NAFLD. In particular, having a high score in the adequacy component was inversely associated with the prevalence of NAFLD. Given that a high score in the adequacy component reflects the recommended intake of foods such as grains, vegetables, fruits, and dairy products, the abundant consumption of these foods may have a protective effect against NAFLD. As a result, our findings suggest that promoting healthy dietary patterns and adherence to Korean dietary guidelines can play a beneficial role in reducing the risk of NAFLD.

Our study findings are in line with those of previous studies showing that healthy dietary patterns are associated with a decreased risk of NAFLD. For instance, previous studies have shown that indices assessing dietary patterns, such as the Healthy Eating Index-2015, Mediterranean Diet Score, and Dietary Approaches to Stop Hypertension, are inversely associated with the risk of NAFLD in the United States (US) [12,14,33,34,35]. Studies have also demonstrated that high-quality diet patterns play beneficial roles in the management and amelioration of NAFLD [36,37]. The concept and scoring system of the KHEI are shared with the Healthy Eating Index developed in the United States [38]. The scoring systems of the KHEI and of the Healthy Eating Index are based on the Healthy Vegetarian and Healthy Mediterranean-Style Dietary Patterns [38]. However, a limitation of the existing literature is that the study populations have primarily been restricted to Western populations, and evidence regarding the association between dietary patterns and NAFLD in East Asian populations, including Korea, is scarce. Therefore, our study is meaningful in confirming a similar inverse association between the Healthy Eating Index and NAFLD, as previously identified in Western populations, now also demonstrated within the Asian population.

The observed association between the KHEI and NAFLD is not surprising since healthy dietary quality is an important factor for the prevention of diseases related to metabolic dysfunction [18,19,20,39]. Reflecting the eating habits of the Korean population, having a high KHEI score is characterized by not skipping breakfast, eating a high number and variety of fruits and vegetables, and consuming carbohydrates, fat, sugar, and sodium in moderation. Maintaining such a healthy dietary pattern is expected to reduce the risk of NAFLD by mitigating cardiometabolic risks such as obesity, DM, and hypertension. For instance, previous studies have reported that individual dietary habits and nutrients, including not skipping breakfast, adequate fiber intake, and consuming dairy products, can potentially decrease the risk of metabolic diseases and NAFLD [3,4,5,40]. Previous studies have consistently shown that skipping breakfast is associated with an increased risk of fatty liver diseases as well as other metabolic abnormalities, including type 2 diabetes and obesity [41,42]. This may contrast with the perception that intermittent fasting diets are often used for the purpose of caloric restriction. However, it has been suggested that individuals who frequently skip breakfast may experience difficulties in appetite regulation, potentially leading to binge eating episodes, which could contribute to weight gain and metabolic dysfunction. A previous study found that breakfast skipping is associated with an elevated level of fasting leptin [43]. Moreover, consuming an adequate amount of carbohydrates in the morning may help alleviate the burden on pancreatic β-cells and improve insulin function [44]. Therefore, having dietary patterns that adhere to the Korean dietary guidelines can contribute to a reduced risk of NAFLD, which is a well-documented hepatic manifestation of metabolic dysfunction.

From a public health perspective, our findings underscore the importance of promoting healthy dietary patterns to mitigate the growing burden of NAFLD in the Korean population. A recent study indicated that, while a gradual improvement in dietary quality has been observed in Korea over the past few decades, a concerning decline in dietary habits among those of younger age and low socioeconomic status persists [45]. This is particularly significant given the notable rise in the prevalence of NAFLD among youth [2]. Our research underscores that pro-active policy interventions aimed at promoting healthier dietary patterns are required within these vulnerable populations. 

Our study had several limitations. First, its cross-sectional design prevented us from establishing a true causal relationship between dietary patterns and NAFLD risk. The temporal sequence of dietary patterns and NAFLD could not be explored. For example, there is a possibility of reverse causation, wherein individuals with NAFLD might adopt healthier dietary patterns to improve their health conditions, potentially mitigating the negative association between the KHEI and NAFLD. Despite the potential underestimation issue, we observed a negative association between the KHEI and NAFLD, demonstrating a dose-dependent pattern. Second, as this was an observational study, we were unable to control for various unmeasured confounders, such as genetic variation and exposure to hepatotoxins, due to the lack of relevant information. Additionally, several health conditions that could potentially confound the effects, including diabetes, hypertension, dyslipidemia, and body mass index, were not considered in the analyses, which may have resulted in an overestimation of the association between the KHEI and NAFLD. Third, the assessment of dietary patterns relied on a 24 h dietary recall, which might not accurately capture long-term dietary habits. Fourth, the classification of NAFLD was based on non-invasive measurements rather than imaging modalities. Although non-invasive measurements like the HSI, the K-NAFLD score, and the ZJU index are widely used in epidemiological studies, relying on these indices for classification can result in misclassification errors. Specifically, the HSI has demonstrated a relatively low sensitivity and positive predictive value in diagnosing fatty liver disease. Therefore, future studies employing imaging modalities or pathologic confirmation are recommended. Fifth, we excluded patients diagnosed with cirrhosis from the analysis. Despite their small number, their cirrhosis could have been attributed to NAFLD. Therefore, excluding them may have led to an underestimation of the association between the KHEI and NAFLD.

Nevertheless, our study had some strengths. The findings were derived from a nationally representative sample of the Korean population, enhancing the generalizability of our results. Additionally, our study was the first, to the best of our knowledge, to reveal an inverse association between the KHEI and NAFLD status. A high KHEI score demonstrated a negative correlation with NAFLD in a dose-dependent manner, emphasizing the significance of maintaining a healthy diet for the prevention or management of NAFLD in the Korean population.

## 5. Conclusions

Our study found that a high KHEI score, characterized by regular breakfast consumption, a high intake of fruits and vegetables, and balanced intake of dietary fat, sodium, and carbohydrates, was inversely associated with the odds of NAFLD. These findings suggest that promoting healthy dietary patterns and adherence to Korean dietary guidelines may be beneficial for the prevention or management of NAFLD and could contribute to a reduction in the growing public health burden of NAFLD.

## Figures and Tables

**Table 1:** Characteristics of the survey participants according to dietary quality.

	Overall	KHEI Score			
		Lowest (Q1: 0–52.8)	Low (Q2: 52.8–62.7)	High (Q3: 62.7–72.0)	Highest (Q4: 72.0–100)
**Sex**					
Male	13,143 (38.5)	3207 (43.8)	3341 (40.4)	3500 (39.2)	3095 (32.1)
Female	21,031 (61.5)	4122 (56.2)	4933 (59.6)	5422 (60.8)	6554 (67.9)
**Age**					
<30	4117 (12.0)	1874 (25.6)	1080 (13.1)	698 (7.8)	465 (4.8)
30–39	5014 (14.7)	1533 (20.9)	1333 (16.1)	1194 (13.4)	954 (9.9)
40–49	6018 (17.6)	1270 (17.3)	1553 (18.8)	1646 (18.4)	1549 (16.1)
50–59	6385 (18.7)	1038 (14.2)	1467 (17.7)	1767 (19.8)	2113 (21.9)
≥60	12,640 (37.0)	1614 (22.0)	2841 (34.3)	3617 (40.5)	4568 (47.3)
**Education level**					
Middle school or below	10,730 (31.4)	1774 (24.2)	2677 (32.4)	3080 (34.5)	3199 (33.2)
High school	11,082 (32.4)	2701 (36.9)	2603 (31.5)	2724 (30.5)	3054 (31.7)
College or above	12,362 (36.2)	2854 (38.9)	2994 (36.2)	3118 (34.9)	3396 (35.2)
**Income level**					
Q1	8164 (23.9)	2251 (30.7)	2171 (26.2)	1998 (22.4)	1744 (18.1)
Q2	8561 (25.1)	1835 (25.0)	2164 (26.2)	2318 (26.0)	2244 (23.3)
Q3	8674 (25.4)	1752 (23.9)	2057 (24.9)	2265 (25.4)	2600 (26.9)
Q4	8775 (25.7)	1491 (20.3)	1882 (22.7)	2341 (26.2)	3061 (31.7)
**Marital status**					
Married	28,619 (83.7)	4999 (68.2)	6826 (82.5)	7883 (88.4)	8911 (92.4)
Unmarried or others	5555 (16.3)	2330 (31.8)	1448 (17.5)	1039 (11.6)	738 (7.6)
**Employment status**					
Employed	14,032 (41.1)	3400 (46.4)	3541 (42.8)	3573 (40.0)	3518 (36.5)
Self-employed	5728 (16.8)	1101 (15.0)	1368 (16.5)	1651 (18.5)	1608 (16.7)
Unemployed	14,414 (42.2)	2828 (38.6)	3365 (40.7)	3698 (41.4)	4523 (46.9)
**Smoking status**					
Past/never smoker	30,274 (88.6)	5982 (81.6)	7214 (87.2)	8000 (89.7)	9078 (94.1)
Current smoker	3900 (11.4)	1347 (18.4)	1060 (12.8)	922 (10.3)	571 (5.9)
**Physical activity**					
No	19,225 (56.3)	4139 (56.5)	4796 (58.0)	5079 (56.9)	5211 (54.0)
Yes	14,949 (43.7)	3190 (43.5)	3478 (42.0)	3843 (43.1)	4438 (46.0)

KHEI: Korean Healthy Eating Index; values are presented as n (weighted %).

**Table 2:** Prevalence of non-alcoholic fatty liver disease according to the study characteristics.

	NAFLD	
	Yes	No
**Total KHEI score**		
Lowest (Q1)	1854 (26.2)	5475 (73.8)
Low (Q2)	2004 (25.1)	6270 (74.9)
High (Q3)	2107 (24.0)	6815 (76.0)
Highest (Q4)	2015 (20.5)	7634 (79.5)
**Sex**		
Male	3497 (29.2)	9646 (70.8)
Female	4483 (19.5)	16,548 (80.5)
**Age**		
<30	782 (19.9)	3335 (80.1)
30–39	1203 (26.4)	3811 (73.6)
40–49	1411 (25.3)	4607 (74.7)
50–59	1644 (25.4)	4741 (74.6)
≥60	2940 (23.1)	9700 (76.9)
**Education level**		
Middle school or below	2787 (25.9)	7943 (74.1)
High school	2630 (24.4)	8452 (75.6)
College or above	2563 (22.6)	9799 (77.4)
**Income level**		
Q1	2143 (26.8)	6021 (73.2)
Q2	2084 (25.5)	6477 (74.5)
Q3	1938 (22.5)	6736 (77.5)
Q4	1815 (21.3)	6960 (78.7)
**Marital status**		
Married	6749 (24.2)	21,870 (75.8)
Unmarried or others	1231 (23.3)	4324 (76.7)
**Employment status**		
Employee	3226 (23.9)	10,806 (76.1)
Self-employed	1465 (26.9)	4263 (73.1)
Unemployed	3289 (22.9)	11,125 (77.1)
**Smoking status**		
Past/never smoker	6819 (22.7)	23,455 (77.3)
Current smoker	1161 (31.7)	2739 (68.3)
**Physical activity**		
No	4715 (25.1)	14,510 (74.9)
Yes	3265 (22.8)	11,684 (77.2)

KHEI: Korean Healthy Eating Index; NAFLD: non-alcoholic fatty liver disease; values are presented as n (weighted %).

**Table 3:** Association between Korean Healthy Eating Index and non-alcoholic fatty liver disease.

	Cases/N	Model 1	Model 2	Model 3
		OR (95% CI)	OR (95% CI)	OR (95% CI)
**Total KHEI score**				
Lowest (Q1)	1854/7329	Reference	Reference	Reference
Low (Q2)	2004/8274	0.95 (0.87–1.03)	0.92 (0.84–1.00)	0.95 (0.87–1.03)
High (Q3)	2107/8922	0.89 (0.82–0.97)	0.85 (0.78–0.92)	0.90 (0.83–0.98)
Highest (Q4)	2015/9649	0.73 (0.67–0.79)	0.71 (0.65–0.78)	0.79 (0.72–0.87)

OR: odds ratio; CI: confidence interval; KHEI: Korean Healthy Eating Index; Model 1: unadjusted model; Model 2: adjusted for sex and age; Model 3: adjusted for sex, age, education level, income level, marital status, employment status, smoking status, physical activity, and survey years.

## Data Availability

The raw data of the KNHANES are available to the public and are accessible on the KNHANES website (https://knhanes.kdca.go.kr/knhanes, accessed on 23 December 2023).

## Supplementary Materials

The following supporting information can be downloaded at: https://www.mdpi.com/article/10.3390/nu16101516/s1, Table S1: Distribution of the subcomponents of the Korean Healthy Eating Index according to the categorization of the total Korean Healthy Eating Index score. Table S2: Supplementary criteria for the classification of non-alcoholic fatty liver disease. Values are presented as weight-adjusted means ± standard deviation. Table S3: Association between each component of the Korean Health Eating Index and NAFLD status (NAFLD: non-alcoholic fatty liver disease; OR: odds ratio; CI: confidence interval). Figure S1: Violin plot and boxplot of the distribution of Korean Healthy Eating Index (KHEI) scores among the study sample. Figure S2: Association between the Korean Healthy Eating Index and its components and non-alcoholic fatty liver disease based on the K-NAFLD score (OR: odds ratio; CI: confidence interval). Figure S3: Association between the Korean Healthy Eating Index and its components and non-alcoholic fatty liver disease based on the ZJU index (OR: odds ratio; CI: confidence interval). Figure S4: Association between the Korean Healthy Eating Index and its components and non-alcoholic fatty liver disease based on the imputed datasets (OR: odds ratio; CI: confidence interval).
