# Supplementary material for: Association between Dietary Quality and Non-Alcoholic Fatty Liver Disease in Korean Adults: A Nationwide, Population-Based Study Using the Korean Healthy Eating Index (2013–2021)
Source: Nutrients. 2024 May 17;16(10):1516. doi: 10.3390/nu16101516 (PMC11124450; doi:10.3390/nu16101516)
Supplement: Supplementary file 1 [file nutrients-16-01516-s001.zip › nutrients-2898732-supplementary.pdf]

Table S1. Distribution of subcomponents of Korean Healthy Eating Index according to the categorization of the total Korean Healthy Eating Index score.

	Scoring system			Overall sample	KHEI score			
	Range	Criteria			Lowest	Low	High	Highest
		Minimum score	Maximum score[a]		(Q1: 0–52.8)	(Q2: 52.8–62.7)	(Q3: 62.7–72.0)	(Q4: 72.0–100)
**Total KHEI score**	0–100			63.6±13.4	45.9±6.8	59.6±2.9	68.7±2.6	80.2±5.2
**Adequacy component**								
**Total adequacy score**	0–55			31.5±10.5	20.1±7.6	28.3±6.3	34.8±5.6	42.8±5.5
Have breakfast	0–10	0 times/week	≥5 times/week	7.3±3.8	4.5±4.1	7.1±3.8	8.4±3.0	9.3±2.1
Mixed grains intake	0–5	0 serving/day	≥0.3 serving/day	2.1±2.2	1.1±1.7	1.9±2.1	2.4±2.2	3.2±2.1
Total fruits intake	0–5	0 serving/day	≥3/2 (men/women) serving/day	2.3±2.2	0.8±1.5	1.8±2.1	2.8±2.1	3.9±1.6
Fresh fruits intake	0–5	0 serving/day	≥1 serving/day	2.5±2.4	0.9±1.8	2.0±2.3	3.0±2.3	4.2±1.7
Total vegetables intake	0–5	0 serving/day	≥8 serving/day	3.5±1.5	2.7±1.6	3.4±1.5	3.8±1.3	4.1±1.2
Vegetables intake excluding Kimchi and pickled vegetables intake	0–5	0 serving/day	≥5 serving/day	3.2±1.6	2.4±1.7	3.0±1.6	3.5±1.5	4.0±1.3
Meat, fish, eggs, and beans intake	0–10	0 serving/day	≥5 serving/day	7.0±3.1	5.7±3.5	6.6±3.1	7.4±2.8	8.4±2.2
Milk and milk products intake	0–10	0 serving/day	≥1 serving/day	3.4±4.4	1.9±3.7	2.6±4.1	3.4±4.4	5.8±4.6
**Moderation component**								
**Total moderation score**	0–30			23.2±5.8	19.8±6.8	23.2±5.6	24.2±4.8	25.7±3.7
Percentage of energy from saturated fatty acid	0–10	>10% of total energy intake	<7% of total energy intake	7.6±3.8	5.5±4.6	7.6±3.8	8.4±3.1	9.1±2.2
Sodium intake	0–10	>6,500 mg/day	≤2,000 mg/day	6.5±3.4	6.5±3.6	6.5±3.5	6.3±3.4	6.8±3.0
Percentage of energy from sweets, beverages, and alcoholic drinks[b]	0–10	>20% of total energy intake	≤10% of total energy intake	9.0±2.5	7.8±3.6	9.1±2.3	9.5±1.6	9.8±0.9
**Energy balance component**								
**Total energy balance score**	0–15			8.9±4.7	6.1±4.7	8.1±4.5	9.8±4.2	11.7±3.3
Percentage of energy intake from carbohydrate	0–5	<50% or >75% of total energy intake	55–65% of total energy intake	2.5±2.1	1.7±2.1	2.2±2.1	2.7±2.1	3.4±1.8
Percentage of energy intake from fat	0–5	<10% or >35% of total energy intake	15–30% of total energy intake	3.3±2.1	2.3±2.3	3.0±2.2	3.6±2.0	4.3±1.4
Energy intake	0–5	<50% or >75% of the EER	75–125% of the EER	3.1±2.2	2.1±2.3	2.9±2.3	3.5±2.1	4.0±1.8

KHEI: Korean Healthy Eating Index; EER: Estimated energy intake requirement.

[a]Criteria for individuals aged 19–64 years. Further information regarding the scoring system and criteria for the other age groups is found in (Yun et al., 2022).

[b]In the 2019-2021 survey year of the KNHANES, this item was modified to evaluate the percentage of energy intake from total sugar, while maintaining consistency with previous standards in terms of minimum and maximum score criteria and scoring methods.

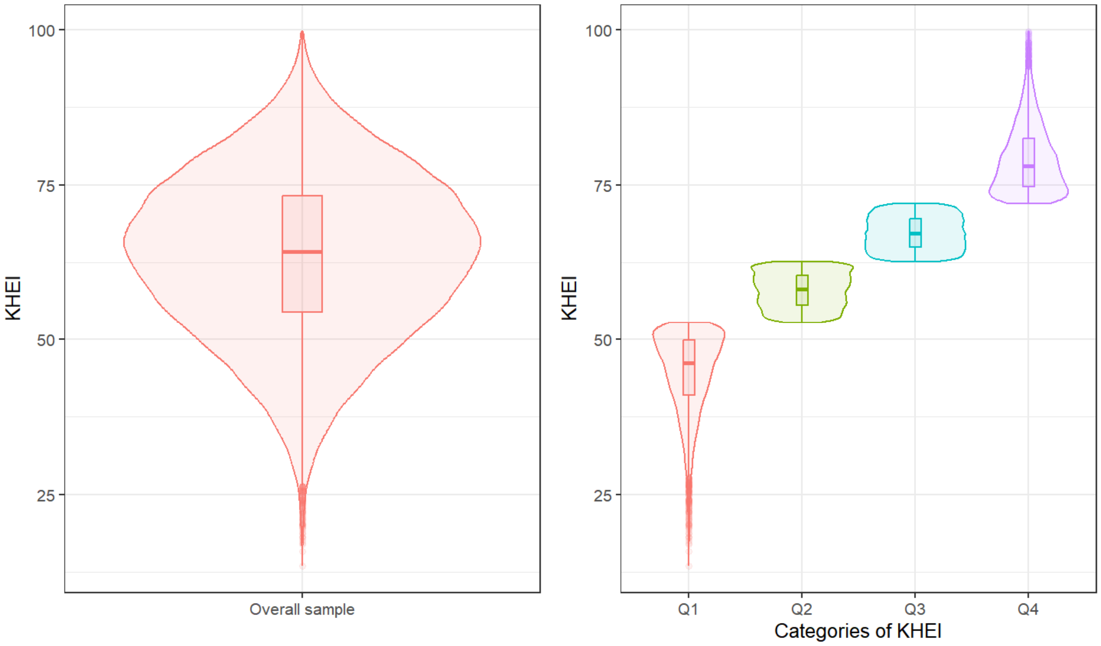

Figure S1. Violin plot and boxplot of the distribution of Korean Healthy Eating Index (KHEI) scores among the study sample

Table S2. Supplementary criteria for the classification of non-alcoholic fatty liver disease.

Measures	Equation	Cutoff
K-NAFLD score[1]	0.913 × sex (2, if female; 1, if male) + 0.089 × WC + 0.032 × (systolic blood pressure + fasting glucose [mg/dL]) + TG (mg/dL) × 0.007 + ALT (IU/L) × 0.105 − 20.929	> 0.884
ZJU index[2]	BMI (kg/m^2) + fasting glucose (mmol/L) + TG (mmol/L) + 3 × (ALT/AST) + 2 (if female)	> 38

WC: Waist circumference; TG: triglycerides; ALT: Alanine aminotransferase; AST: Aspartate aminotransferase

Table S3. Association between each component of the Korean Health Eating Index and NAFLD status (NAFLD: non-alcoholic fatty liver disease; OR: odds ratio; CI: confidence interval).

	NAFLD
	**OR (95% CI)**
**Adequacy component**	
Breakfast (0–10)	0.97 (0.96–0.98)
Mixed grains (0–5)	1.05 (1.02–1.08)
Total fruit (0–5)	0.93 (0.87–1.00)
Fruit, excluding juice (0–5)	1.01 (0.95–1.09)
Total vegetables (0–5)	0.97 (0.93–1.02)
Vegetable, excluding kimchi and pickles (0–5)	1.05 (1.00–1.10)
Meat, fish, eggs, and beans (0–10)	0.97 (0.94–1.01)
Milk and dairy (0–10)	0.96 (0.93–0.99)
**Moderation component**	
Saturated fatty acid (0–10)	1.00 (0.97–1.04)
Sodium (0–10)	0.94 (0.90–0.97)
Empty calorie foods (0–10)	1.01 (0.98–1.05)
**Balance component**	
Carbohydrate (0–5)	1.00 (0.95–1.04)
Fat (0–5)	1.00 (0.96–1.05)
Total energy (0–5)	0.96 (0.93–1.00)

Unit: 1 standard-deviation increase for each item

[1]Jeong S, Kim K, Chang J, Choi S, Kim SM, Son JS, Lee G, Kim W, Park SM. Development of a simple nonalcoholic fatty liver disease scoring system indicative of metabolic risks and insulin resistance. Ann Transl Med. 2020 Nov;8(21):1414.

[2]Wang J, Xu C, Xun Y, Lu Z, Shi J, Yu C, Li Y. ZJU index: a novel model for predicting nonalcoholic fatty liver disease in a Chinese population. Sci Rep. 2015 Nov 16;5:16494.

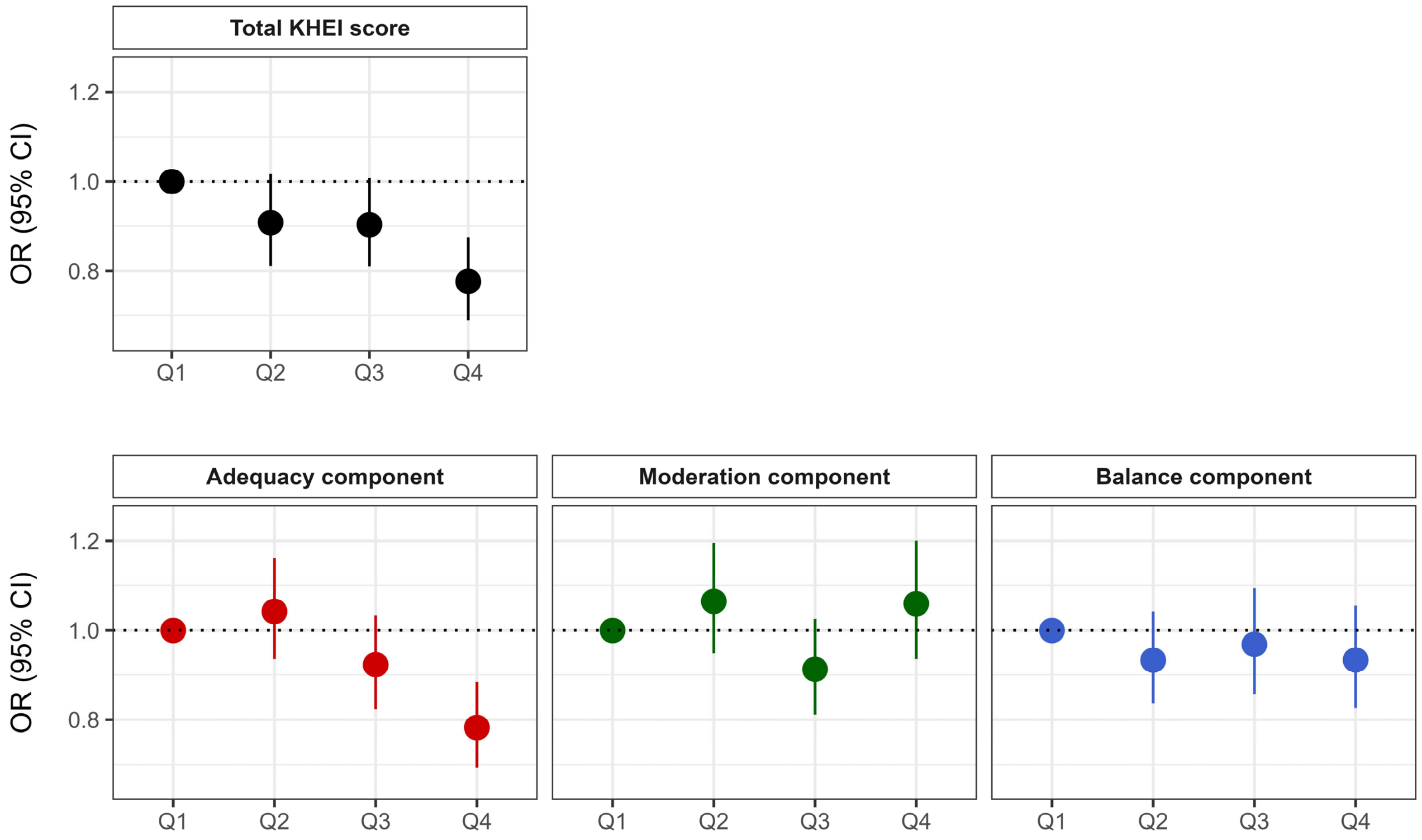

Figure S2. Association between Korean Healthy Eating Index and its components and non-alcoholic fatty liver disease based on the K-NAFLD score (OR: odds ratio; CI: confidence interval).

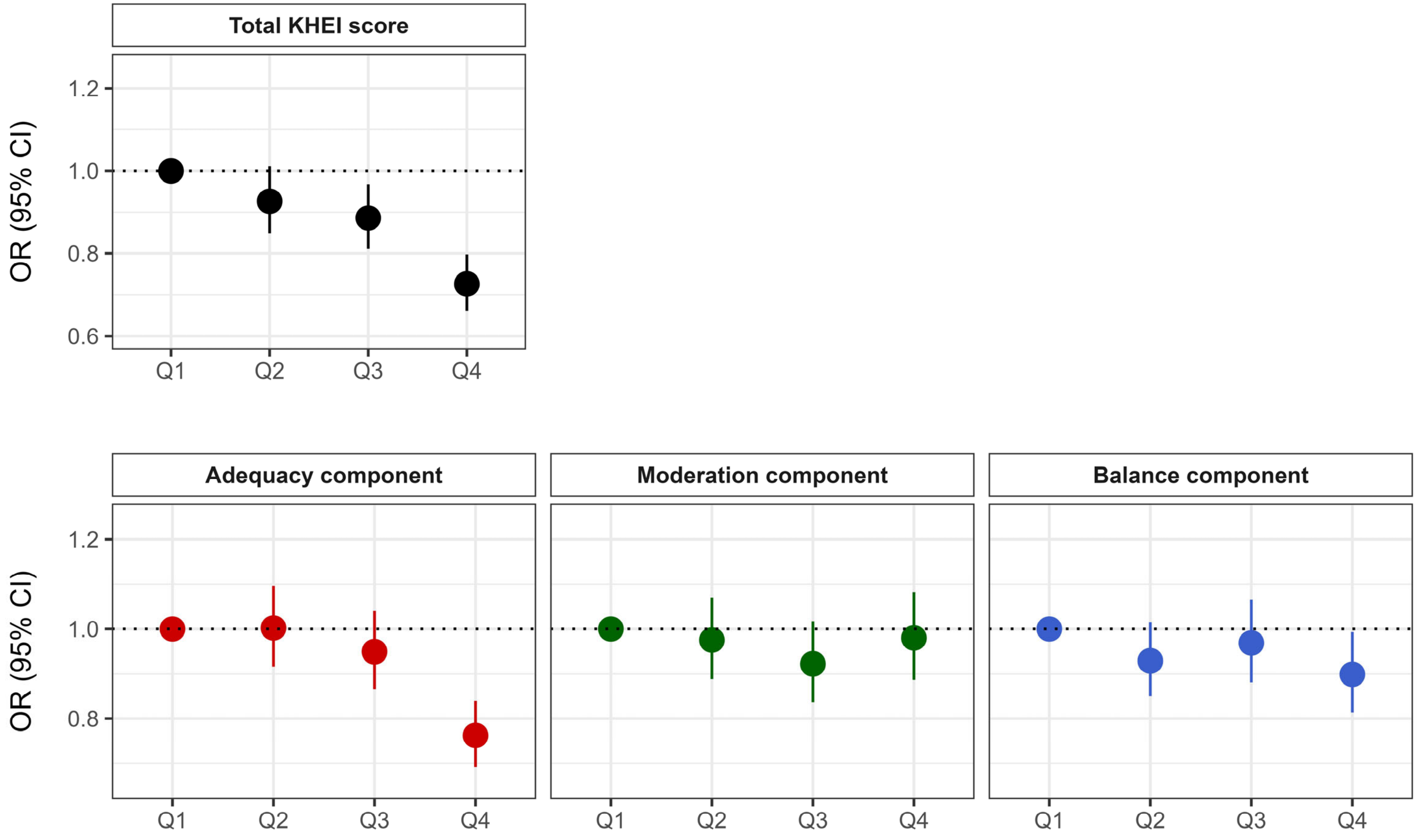

Figure S3. Association between Korean Healthy Eating Index and its components and non-alcoholic fatty liver disease based on the ZJU index (OR: odds ratio; CI: confidence interval).

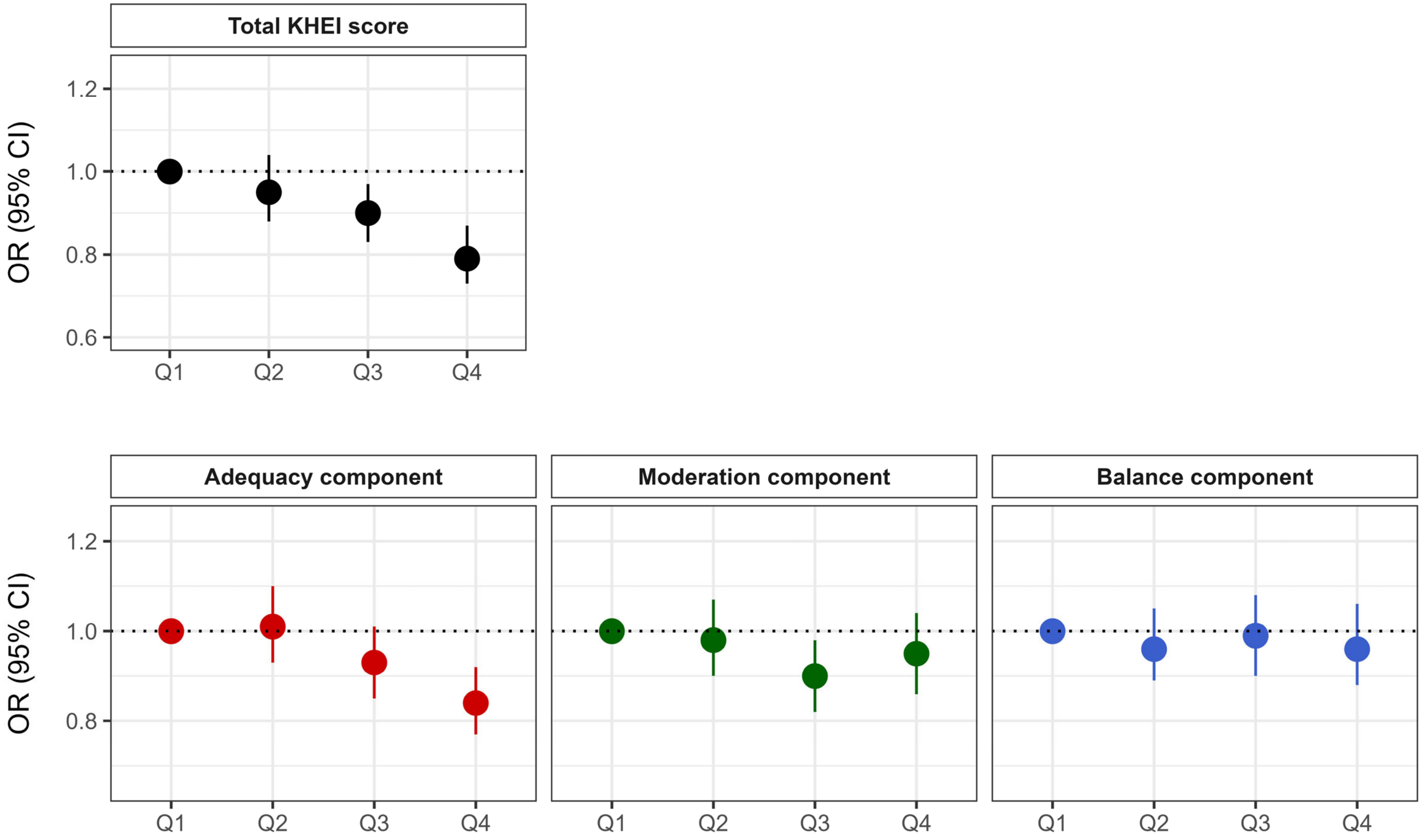

Figure S4. Association between Korean Healthy Eating Index and its components and non-alcoholic fatty liver disease based on the imputed datasets (OR: odds ratio; CI: confidence interval).

References

Yun, S., Park, S., Yook, S.M., Kim, K., Shim, J.E., Hwang, J.Y., Oh, K., 2022. Development of the Korean Healthy Eating Index for adults, based on the Korea National Health and Nutrition Examination Survey. Nutr Res Pract 16:233-47.
